# Characterization of Female External Genitalia and Eggs of Four South American Species of the *Triatoma* Laporte, 1832 Genus (Hemiptera: Reduviidae: Triatominae)

**DOI:** 10.3390/insects12060537

**Published:** 2021-06-10

**Authors:** Tiago Belintani, Jader Oliveira, Heloisa Pinotti, Kaio Cesar Chaboli Alevi, Juliana Damieli Nascimento, Estela Sasso-Cerri, Cleber Galvão, João Aristeu da Rosa

**Affiliations:** 1 Institute of Biology, Campinas State University (Unicamp), Block O, Bertrand Russel Avenue, Campinas 13083-865, São Paulo, Brazil; tiagobellintani@gmail.com (T.B.); judamieli@gmail.com (J.D.N.); 2 Laboratory of Entomology in Public Health, Department of Epidemiology, Faculty of Public Health, University of São Paulo, Av. Arnaldo 01246-904, São Paulo, Brazil; jdr.oliveira@hotmail.com; 3 School of Pharmaceutical Sciences, São Paulo State University (Unesp), Araraquara-Jaú Highway, km 1, Campos Ville, Araraquara 14800-903, São Paulo, Brazil; helopinotti@hotmail.com (H.P.); kaiochaboli@hotmail.com (K.C.C.A.); joaoaristeu@gmail.com (J.A.d.R.); 4 Department of Morphology, Genetics, Orthodontics and Pediatric Dentistry, Dental School, São Paulo State University (Unesp), Rua Humaitá, 1680, Araraquara 14801-903, São Paulo, Brazil; estela.sasso@unesp.br; 5 National and International Reference Laboratory in Taxonomy of Triatomines, Oswaldo Cruz Institute, Fiocruz, Av. Brasil, 4365, Manguinhos 21040-360, Rio de Janeiro, Brazil

**Keywords:** Triatominae, *Triatoma*, morphology, morphometry, Chagas diseases

## Abstract

**Simple Summary:**

We present a morphological and morphometric study with *T. garciabesi*, *T. guasayana*, *T. patagonica,* and *T. sordida sensu stricto* species within the *Triatoma* genus. This group of species is important for the eco-epidemic scenario of Chagas disease in the Americas; their species have morphological, biological, and behavioral similarities that make diagnosis difficult. For the first time, the description of the female external genitalia by scanning electron microscopy (SEM), a character that has helped with the delimitation of species and genera in Triatominae, is published, in addition to presenting an extensive study with eggs, covering morphology and morphometry. The study with eggs is an important tool in taxonomic studies of the subfamily. In addition to taxonomic contributions, it was possible through the descriptions to corroborate the validity of *T. garciabesi* and confirm the current classification of these species.

**Abstract:**

*Triatoma* is the most diversified and one of the most important genera from an epidemiological perspective. Given the difficulty in identifying some species of the *Triatoma* genus, morphological, histological, and morphometric studies were performed to provide new characters that make it possible to differentiate *T. garciabesi*, *T. guasayana*, *T. patagonica,* and *T. sordida sensu stricto*, triatomines that overlap geographically and have vector potential. Through the external female genitalia, as well as morphology, morphometry, and histology of eggshells, it was possible to discriminate the four species. In addition, this study reinforces the taxonomic validity of *T. garciabesi* and provides new data for discussion on systematic issues of *T. guasayana* and *T. patagonica*.

## 1. Introduction

Chagas disease vectors belong to the subfamily Triatominae, which includes 156 extant and three fossil species [1,2,3]. Triatomines occur mainly in the Americas, with few exceptions outside the continent [4]. Among the genera of Triatominae, *Triatoma* Laporte, 1832, is the most representative in species and relevant to the eco-epidemiological scenario of Chagas infection [5]. The diversity in *Triatoma* reflects in inconclusive phylogenies, the genus has at least two paraphyletic groups [6]. In addition to the systematic problems inherent to *Triatoma*, phylogenies with the Tribe Triatomini, recover unnatural groups [6], condition that keeps the discussion about the evolutionary origin of the group [4]. However, the integration of genetic and phenetic characters has helped to elucidate the evolutionary history of the group, but new revisions are necessary [4,7,8]. There are also systematic problems in the organization of complexes and subcomplexes of *Triatoma* [8]. Morphological, biological and behavioral similarities are common in the genus, as in the species *Triatoma garciabesi* Carcavallo, et al. 1967; *Triatoma guasayana* Wygodzinsky and Abalos, 1949; *Triatoma patagonica* Del Ponte, 1929, and *Triatoma sordida* (Stål, 1859) evaluated in this study.

Traditionally, the four species mentioned above formed the *T. sordida* subcomplex proposed by Schofield and Galvão [9]. Currently, after the proposed reorganization by means of chromosomal deposition of the main ribosomal DNA (rDNA) [8] the species *T. garciabesi*, *T. sordida sensu stricto* and *Triatoma rosai* Alevi et al. [3] (*T. rosai* recently described was for a period considered a polymorphic population of *T. sordida* with distribution in Argentina) remain in the *T. sordida* subcomplex, while *T. guasayana* and *T. patagonica* were transferred in the *T. rubrovaria* subcomplex. The new organization was corroborated by phylogenetic study with genetic and morphometric markers [7]. 

These triatomines have morphological and behavioral similarities, in addition, they overlap in some geographic ranges with reports of hybrid specimens [10]. The four species are currently restricted to South America, *T. sordida sensu stricto* has a wide geographical distribution and can be found in Argentina, Bolivia, Brazil, and Paraguay, *T. garciabesi* and *T. patagonica* are restricted to Argentina, while *T. guasayana* occurs in Argentina, Bolivia, and Paraguay [5,11,12]. 

*Triatoma garciabesi* is very similar to *T. sordida*, evident in the morphological, behavioral, and biogeographic similarities [6,13]. The dignity of *T. garciabesi* is complex and errors in identification are frequent [11]. *Triatoma guasayana* has morphological similarity to *T. sordida,* mainly in the early stages of development [14,15], however morphometric, cytogenetics and phylogenetic studies support the taxa as distinct [7,11,16]. *Triatoma patagonica* is distributed in the Southern most region of South America, this naturally wild triatomine has epidemiological relevance and can inhabit artificial environments, such as chicken coops and pens [12]. The species shows greater morphological differences [11,16,17] and genetic differences [7,18,19] in relation to *T. garciabesi*, *T. guasayana*, and *T. sordida*.

Initially described as *Conorhinus sordidus* (Stål, 1859)*, Triatoma sordida* is widely distributed in South American countries [11,18]. Forattini [20] suggests that the species has focus of endemism in the central region of Brazil. The author discusses the preference and tolerance for high temperatures and dry climates, a relevant factor for dispersal of the species. Studies with *T. sordida sensu stricto* show enzymatic, genetic [5,11,21], morphological and morphometric diversity [5,16,22]. The first record of *T. sodida* variability goes back to 1951 [14]. However, the Brazilian populations of *T. sordida sensu stricto* have little genetic variability [23,24,25]. The terminology used in this study for *T. sordida sensu stricto* refers to the suggestion of Panzera et al. [11], the term describes species distributed throughout Brazil, central Paraguay, Chaco, and eastern Bolivia. 

Due to the importance of correct vector identification, this study evaluates morphological and morphometric characters that group and distinguish *T. garciabesi, T. guasayana, T. patagonica,* and *T. sordida sensu stricto*. In addition, we present two new characters for the studied species, the female external genitalia, and the histological profile of the exochoria. 

## 2. Materials and Methods

### 2.1. Taxon Sampling 

**Figure 1:**
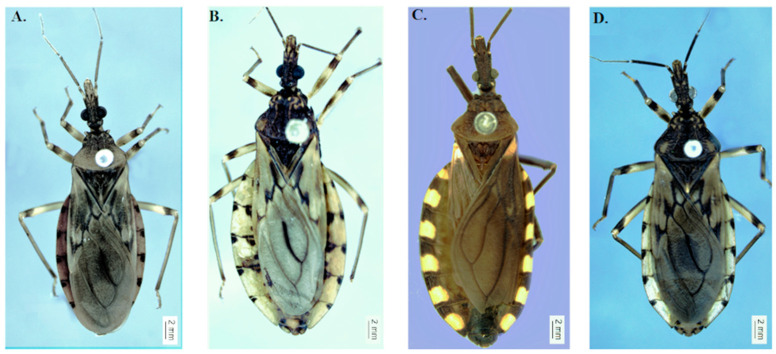
Species analyzed in this study. (**A**) *T. garciabesi*, (**B**) *T. guasayana*, (**C**) *T. patagonica*, and (**D**) *T. sordida sensu stricto*.

The specimens used in this work were obtained from the Triatominae Insectary of São Paulo State University Julio de Mesquita Filho, School of Pharmaceutical Sciences, Araraquara, São Paulo, Brazil (https://www2.fcfar.unesp.br/#!/triatominae accessed on 1 January 2021) accessed on 10 October 2020 and from the Triatomine Collection of the Oswaldo Cruz Institute (http://ctioc.fiocruz.br/ accessed on 1 January 2021) accessed on October 2020. The specimens were identified from descriptions and identification keys [13,17]. In this study we used: *T. garciabesi* (Rivadavia, Province of Medonça, Argentina), *T. guasayana* (Cochabamba, Department of Cochabamba), *T. patagonica* (Jachal, Province of San Juan, Argentina), and *T. sordida sensu stricto* (Seabra, State of Bahia, Brazil) (Figure 1). 

### 2.2. Morphological Studies

#### 2.2.1. Morphologic Study of External Female Genitalia and Eggshells

The images female external genitalia (n = 5) and the eggshells (n = 5) of the four species were produced by scanning electron microscopy (SEM) according to Rosa et al. [25]. After death by freezing in −20 °C, the specimens were dissected from the sixth segment to acquire the final portion of the abdomen, with the female external genitalia. Subsequently, the dissected structures were washed, dehydrated with an alcohol series, and oven dried for 30 min at 50° C. The female external genitalia were examined in the dorsally, posteriorly, and ventrally. The eggshells were also subjected to washing, dehydration, and drying. Subsequently, they were fixed on aluminum stubs. The samples of the female external genitalia and the eggshells were by sputtering with gold for 2 min at 10 mA, as described in Rosa et al. [25]. Subsequently, the female external genitalia (dorsal, posterior, and ventral views) and the eggshells (full and exochorion aspect) were examined with a Topcon SM 300 SEM at the Institute of Chemistry at Unesp, Araraquara, São Paulo, Brazil. The images obtained were processed (background, contrast, brightness) in the GNU Image Manipulation Program v2.0.2 (GIMP) software free and open-source image editor, subsequently the structures were described, and the descriptions were compared.

#### 2.2.2. Histological Analysis 

The eggshell histological procedures were conducted at the Laboratory of Histological Processing of the Dental School, Unesp, Araraquara, São Paulo, Brazil following a protocol adapted from Obara et al. [26]. Five hatched eggs of each species were selected, and the shells were washed in acetone using ultrasound (40 kHz for 3 min) and dried at 40 °C for 30 min. The eggshells were transferred to small plastic cassettes and submitted to histological processing in a “MORSE” descaling solution (formic acid + sodium citrate) for 15 min. With the aid of a scalpel, the shells were split in half under a surgical magnifying glass. The fragments were washed in running water for 1 h, dehydrated in a graded ethanol series (2 h), diaphanized in xylene for 1 h, and embedded in liquid paraffin at 60 °C (2 h). Subsequently, the samples were cut with a LEICA RM2145^®^ microtome, and 5 µm-thick sections were stained with Harris’ Hematoxylin (10 min) and Eosin (5 min). The stained sections were mounted with Permount mounting medium and observed under a Leica microscope © Leitz DM RXE with a digital camera. 

### 2.3. Morphometric Study

#### Morphometric Analysis of Eggshells 

The total length and perimeter of the opercular opening of the eggs were measured based on classical morphometric methods [27,28]. For eggshell morphometry, 50 eggs were selected from *T. garciabesi, T. guasayana, T. patagonica,* and *T. sordida sensu stricto.* Variations were estimated by ANOVA and Tukey’s post-hoc test (*p* < 0.05%) and were computed using GraphPad Prism (Graphpad Software v. 8.00 for Windows, San Diego, CA, USA). 

## 3. Results

### 3.1. Morphological Analyses 

#### 3.1.1. Female External Genitália

The female external genitalia of *T. garciabesi*, *T. guasayana*, *T. patagonica*, and *T. sordida sensu stricto* are described for the first time. Among the species studied, the female external genitals have different characters in the three positions evaluated (dorsal, ventral, and posterior), allowing specific identification. The main differences in female external genitalia are compiled in Table 1.

#### 3.1.2. Dorsal View of Female External Genitalia 

**Figure 2:**
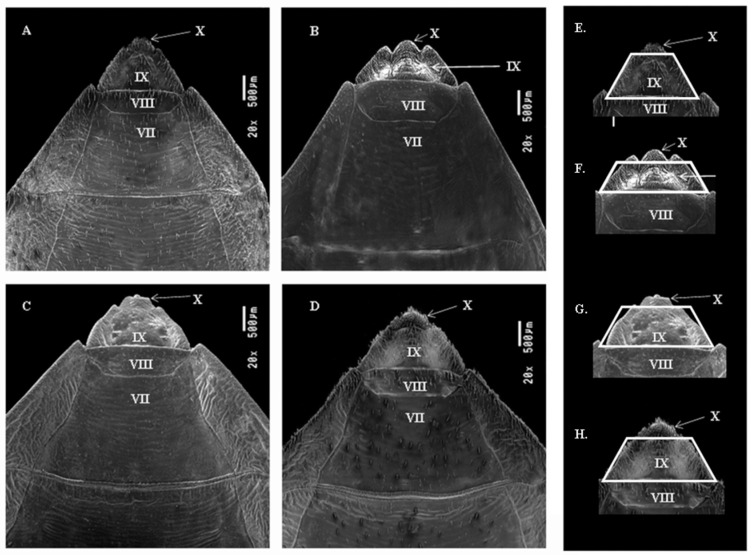
Dorsal view of the female external genitalia of the four species by scanning electron microscopy (SEM). (**A**) *T. garciabesi*, (**B**) *T. guasayana*, (**C**) *T. patagonica*, and (**D**) *T. sordida sensu stricto*, (**E**) *T. garciabesi*, (**F**) *T. guasayana*, (**G**) *T. patagonica*, and (**H**) *T. sordida stricto sensu*.

The borders (with a pair) between the segment VII and the connective are straight in *T. garciabesi* and *T. guasayana* (Figure 2A,B), in *T. patagonica* and *T. sordida sensu stricto* the lower portion is slightly curved (Figure 2C,D).

Posterior portions (with a pair) of the VII segment at the limit with the VIII segment and connexiva end as triangular tips in *T. garciabesi*, *T. guasayana*, *T. patagonica,* and *T. sordida sensu stricto* (Figure 2). The VIII segment has a trapezoid shape in the four species, in *T. guasayana* the lower part is curved in the others the angles are straight. 

**Figure 3:**
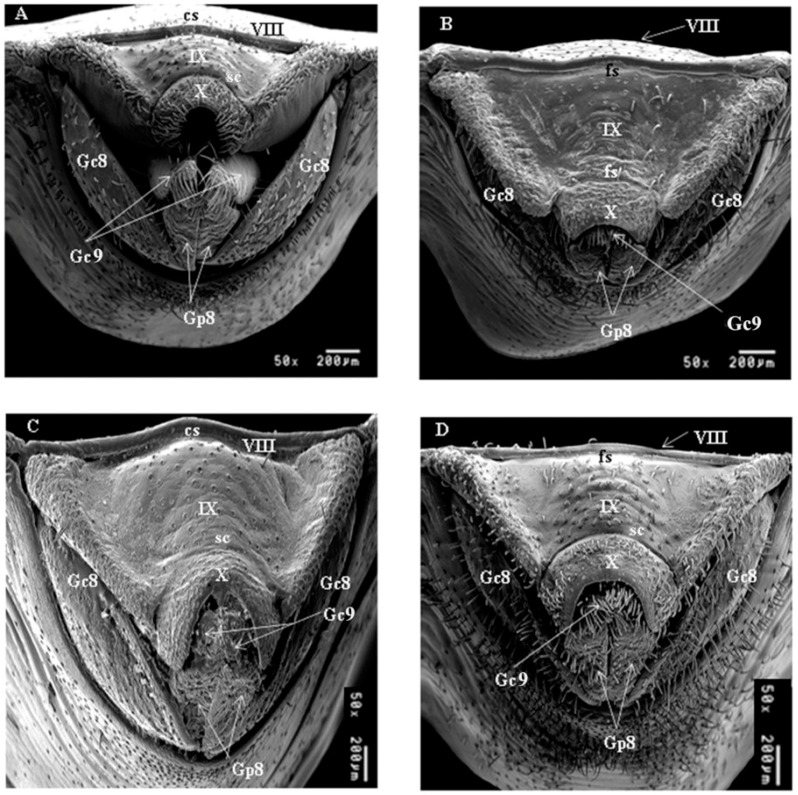
Posterior view of the female external genitalia of the four species by SEM. (**A**) *T. garciabesi*, (**B**) *T. guasayana*, (**C**) *T. patagonica*, and (**D**) *T. sordida sensu stricto*. Subtitles: Gc8 and Gc9: gonocoxites, 8, Gp9: gonapophyses, VII, VIII, tergite, IX, X segments, cs: convex shape, sc: semicircular shape and fs: flat shape.

Segment IX has a trapezoidal shape in the four species, but in *T. guasayana* the region is lower and wider (Figure 2E–H) in, *T. patagonica* is slightly curved on the sides (Figure 3). Posterior portions (with a pair) of the IX segment in *T. guasayana* have the shape of rounded tips (Figure 2B). The posterior portion of segment X is obtuse in *T. garciabesi, T. guasayana* and *T. sordida sensu stricto* and flat in *T. patagonica* (Figure 2). 

#### 3.1.3. Posterior View of Female External Genitalia 

In *T. patagonica* and *T. garciabesi* the border of region dividing the VIII and IX segments is convex (cs) and flat in *T. guasayana* and *T. sordida sensu stricto* (Figure 3). In the four species the limits between the IX and X segments are open on the sides and closed in the central area (Figure 3); moreover, in *T. patagonica* the region is very curved compared to the other species. The lateral borders of the IX are curved and open close to the VIII in *T. garciabesi, T. guasayana*, and *T. sordida sensu stricto*, unlike *T. patagonica* that close. The X segment are different between species, the border between IX and X are determinant for the differences, in *T. garciabesi* in *T. sordida sensu stricto* it is semicircular (sc) in *T. gasayana* it is slightly flat (fs) and in *T. patagonica* it is considerably curved elongated (Figure 3). According to the images obtained, *T. sordida sensu stricto* presents a greater number of bristles in relation to the others.

#### 3.1.4. Ventral View of Female External Genitalia 

**Figure 4:**
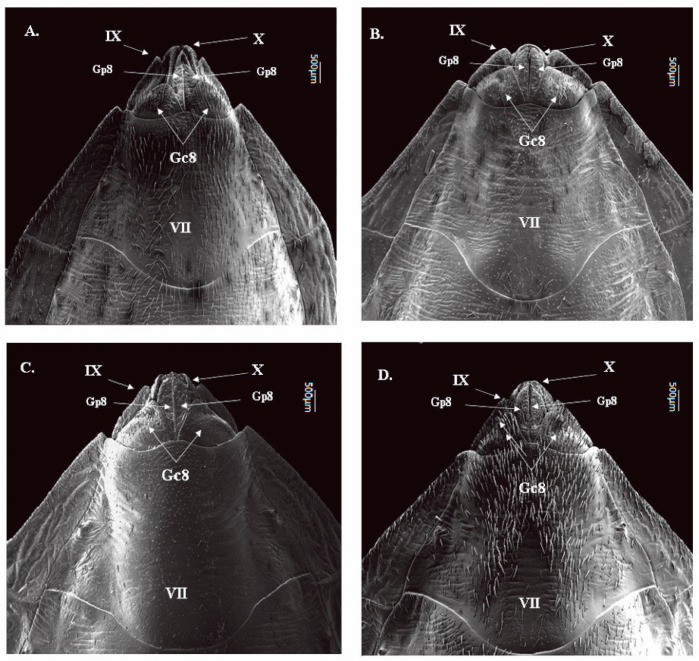
Ventral view of the female external genitalia of the four species by SEM. (**A**) *T. garciabesi*, (**B**) *T. guasayana*, (**C**) *T. patagonica*, and (**D**) *T. sordida sensu stricto*.

In the four species, the border of the VII segment with Gc8 and Gp8 is concave on the sides (with a pair) and convex in the central portion (Figure 4). Posterior portions of the connexiva of the VII segment end in pointed tips (with a pair) in *T. garciabesi, T. guasayana*, and *T. patagonica* (Figure 4A–C) in *T. sordida sensu stricto* is not present (Figure 4D). The Gc8 shape of *T. guasayana and T. patagonica* is triangular and in *T. garciabesi* and *T. sordida sensu stricto* it is triangular and obtuse. Gp9 is similar in all species, however in *T. sordida sensu stricto* it is smaller. (Figure 4). In *T. garciabesi, T. patagonica and T. sordida sensu stricto* the IX forms an isosceles triangle, while in *T. patagonica* the shape refers to a slightly obtuse equilateral triangle. (Figure 4B). The IX is apparent in all species: *T. garciabesi* the segment shape is triangular and well angled, *T. gasayana* the segment is very visible and has the obtuse final portion, *T. patagonica* the region is triangular, and the outer edges are curves, and *T. sordida sensu stricto* the segment is triangular with many bristles, these bristles also extend throughout the central region of VII, X, and through Gc8.

#### 3.1.5. Morphology of Eggs 

**Figure 5:**
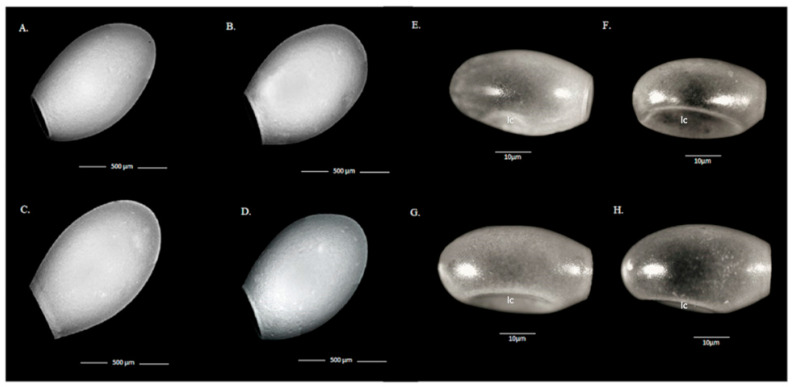
Eggshells’ general view by scanning electron microscopy (x50) and by light microscopy (x100). (**A**) *T. garciabesi*, (**B**) *T. guasayana*, (**C**) *T. patagonica*, and (**D**) *T. sordida sensu stricto*, (**E**) *T. garciabesi*, (**F**) *T. guasayana*, (**G**) *T. patagonica*, and (**H**) *T. sordida sensu stricto*. Longitudinal chamfers (lc).

Through SEM and light microscopy, it was possible to describe the architecture of the ultrastructure and the general appearance of the eggs of the four species. In general, the eggs analyzed are ellipsoidal in the four species studied (Figure 5). The four species show longitudinal chamfers that are very apparent in *T. guasayana*, *T. patagonica*, and *T. sodida* and less apparent in *T. garciabesi* (Figure 5).

**Figure 6:**
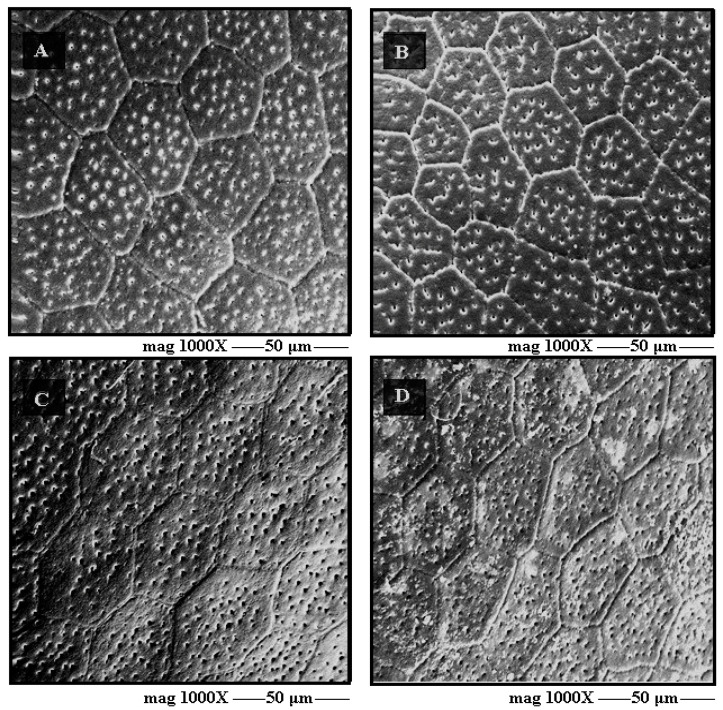
Exochorion detail via scanning electron microscopy (x1000). (**A**) *T. garciabesi*, (**B**) *T. guasayana*, (**C**) *T. patagonica*, and (**D**) *T. sordida sensu stricto*.

The surface of exochoria in SEM is different between species. In general, the predominant shapes are pentagons, hexagons, and heptagons. In *T. garciabesi*, *T. guasayana* and *T. sordida sensu stricto* the shapes of the exochorion surface are varied without a defined pattern, while in *T. patagonica* the shapes are hexagonal. The profile of the limiting lines is different between species, it can be seen that in *T. garciabesi* and *T. guasayana* the lines are deep and define a quilted appearance for the exochorial structures, while in *T. patagonica* and *T. sordida sensu stricto* the lines are shallow and offer a flat appearance for exochorion (Figure 6). In the four species, numerous perforations can be observed scattered throughout the exochorion (Figure 6).

#### 3.1.6. Histological Analysis of Eggs 

**Figure 7:**
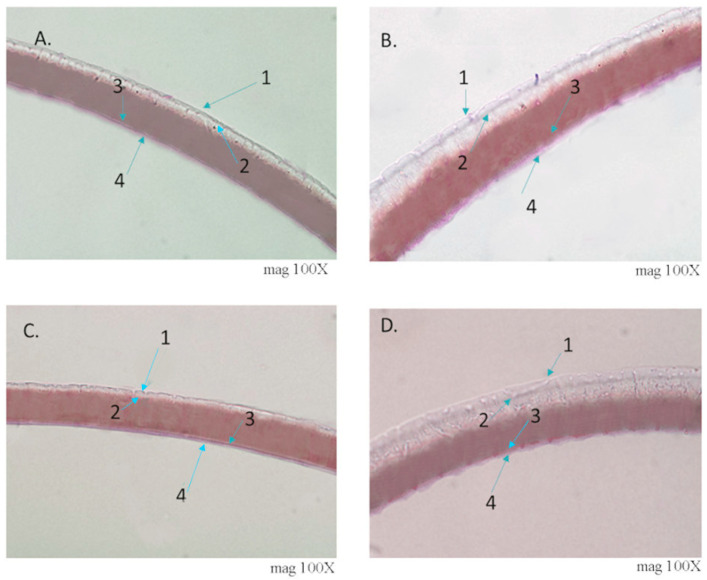
Photomicrographs of sections of eggs chorion stained with Harris’ Hematoxylin and Eosin at x100 magnification. (**A**) *T. garciabesi*, (**B**) *T. guasayana*, (**C**) *T. patagonica*, and (**D**) *T. sordida sensu stricto*. 1. stiff exochorion, 2. soft exochorion, 3. stiff endochorion, and 4. soft endochorion.

The egg sections of the four species were analyzed using optical microscopy (Figure 7). Photomicrographs make it possible to identify four layers of tissue. The internal tissues: soft endochorion and the stiff endochorion (Figure 7) and the external tissues, the stiff exochorion and the soft exochorion (Figure 7).

Different textures can be observed between the studied tissues: the endochoria of *T. garciabesi* and *T. patagonica* (internal part) is smooth (Figure 7A,C), while in *T. sordida sensu stricto* and *T. guasayana* is wavy (Figure 7B,D). The profile of the egg exochoria is wavy in the four species, probably because of the hexagonal and pentagonal ornamentation of the exochoria. In the photomicrographs it is possible to perceive the differences in the thickness of the tissues: *T. garciabesi* and *T. patagonica* are thin and relationship and *T. guasayana* and *T. sordida sensu stricto* thick. There are also differences in the thickness of the stiff exochorion, in guasayana and T. sordida are thick in *T. garciabesi* and *T. patagonica* are less apparent.

### 3.2. Morphometric Study

#### Morphometry of Eggs

**Figure 8:**
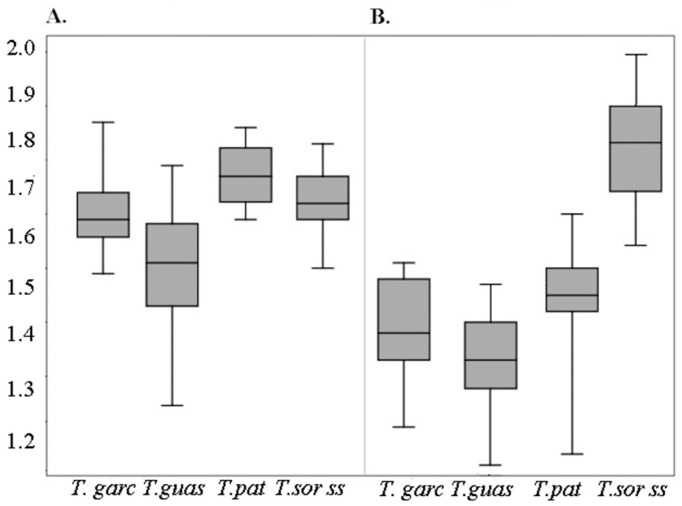
Box plot showing variation of length of eggs (**A**) and perimeter opening of the operculum (**B**). *T. garciabesi* (T. garc), *T. guasayana* (T. guas), *T. patagonica* (T. pat), and *T. sordida stricto sensu* (T. sord ss).

The two measured parameters show the metric variability between eggs of the four studied species (Figure 8). Regarding the total length of the eggs, *T. patagonica* presents the largest average size with 1.77 ± 0.05 mm followed by *T. sordida sensu stricto* 1.72 ± 0.05 mm. *T. garciabesi* with 1.70 ± 0.09 mm and *T. guasayana* 1.61 ± 0.09 mm have a lower mean total length of the eggs. *T. garciabesi* and *T. sordida sensu stricto* have statistically similar total egg length (Table 2). Therefore, the relation according to the egg length is: *T. patagonica* > *T. sodida sensu stricto* > *T. garciabesi > T. guasayana* (Figure 8).

The perimeter of the opening of the operculum showed greater capacity to discriminate eggs among the four species. The largest mean size of the perimeter of the opening of the operculum was *T. sordida sensu stricto* 1.83 ± 0.10 mm, followed by *T. patagonica* with 1.5 5 ± 0.07 mm, the averages of *T. garciabesi* and *T. patagonica* were lower 1.47 ± 0.10 mm and 1.42 ± 0.08 mm, respectively (Table 2). The morphometric relationship of the perimeter opening of the operculum from the largest to the smallest is: *T. sordida sensu stricto* > *T. patagonica > T. garciabesi* > *T. guasayana*.

The variables of length and perimeter of the operculum forma the estimates for the levels of significance. The four species show significant differences between the averages. However, *T. garciabesi* and *T. sordida sensu stricto* do not show differences in length averages (Table 3).

## 4. Discussion

In the subfamily Triatominae, the *Triatoma* genus currently has the largest number of species, with its complexes and subcomplexes [9] clearly being the most diverse of the subfamily [6]. Discussions about species of *Triatoma* deserve attention, especially in view of the eco-epidemic scenario in which are inserted [29]. In this study, we present new taxonomic characters for four species of the genus: *T. garciabesi*, *T. guasayana*, *T. patagonica*, and *T. sordida sensu stricto*, in addition, the female external genitalia and histology of eggs are presented with new diagnostic characters.

Although the recent phylogenies [7] show a distancing of *T. guasayana* and *T. patagonica* from *T. garciabesi* and *T. sordida sensu stricto*, these species have similar morphology and biology, in addition they overlap geographically by several South American regions where they have vectorial potential and consequently deserve attention [10,11,13].

*Triatoma garciabesi* has been described from specimens captured in central Argentina [30] but synonymized with *T. sordida* by Lent and Wygodzinsky [17]. Through morphological characters of the head, male genitalia, izoenzymes, and cytogenetics, the species was revalidated in 1998 [13]. In the present study, the results allowed to differentiate *T. garciabesi* from the other studied species, despite the morphological similarities shared mainly with *T. sordida sensu stricto*, the results corroborate the revalidation. We demonstrate that it is possible to discriminate the species by the characters of the external female genitalia and by the morphometry of the eggs, however the morphology of the eggs presents similarities that make the diagnosis difficult.

We demonstrate that it is possible to differentiate the species based on the morphological differences of the female external genitalia evaluated in dorsal, posterior, and ventral view. The dorsal and ventral position has a greater number of characters useful for discriminating these triatomines. In the dorsal position, IX and X have different shapes, being similar in *T. garciabesi* and *T. sordida stricto sensu.* In the ventral view, Gc8 are determinants for taxonomy of the studied species. The characterization of the female external genitalia by SEM in Triatominae allows diagnosis at the level of gender [31] and species, in this case assisting in the diagnosis of nearby taxons [2,25,32]. The female external genitalia were used in a study with 10 species of the *T. brasiliensis* subcomplex [9] corroborating the diagnosis of the studied species, in addition to helping in inferring the group’s systematic relationships [32].

Eggs of triatomines possess characteristics that are useful in taxonomic studies [27,33]. In this study, the morphological and morphometric data of the eggs proved to be useful to discriminate the four species studied. The results show that the eggs of *T. patagonica* are the ones that show the greatest morphological and morphometric differences, *Triatoma guasayana* presents eggs of shorter length when compared to the studied species in addition to morphological differences. *Triatoma garciabesi* and *T. sordida sensu stricto* have morphology and morphometric similarities in eggs, however the histological profile is different.

By comparing the results of this study with previous publications, it was possible to determine morphological relationships between the eggs of the species studied with others of *Triatoma*. The characteristics described for eggs of *T. guasayana* and *T. patagonica* allow to show that they present morphological similarities with eggs of species of the *T. rubrovaria* subcomplex. The results show that the eggs of *T. patagonica* are the ones that present the greatest morphological and morphometric differences, the elongated profile is similar to the published descriptions for eggs of the species *Triatoma rubrovaria* (Blan-chard, 1846) and *Triatoma klugi* Jurbergi, Lent and Galvão, 2001 [34,35]. The approximate average length of the *T. guasayana* eggs are like those described for *Triatoma circummaculata* (Stål, 1859) and *Triatoma carcavalloi* Jurberg, Rocha and Lent, 1998, species of the *T. rubrovaria* subcomplex [34,36]. It was also possible to show that the morphology of *T. sordida sensu stircto* eggs is similar to the morphology of *T. matogrossensis* Leite e Barbosa, 1953; *T. vandae* Carcavallo et al., 2002 and *T. jurbergi* Carcavallo, Galvão and Lent, 1998 [36,37,38]. *Triatoma matogrossensis*, *T. vandae*, and *T. jurbergi* were recently inserted together with the *T. sordida* subcomplex [7,8].

The proximity of *T. guasayana* and *T. patagonica* it was first discussed by Abalos and Wygodzinsky [14] classifying both as allopatric species. Usinger et al. [39] suggest the proximity between *T. guasayana* and *T. patagonica* to *T. sordida.* At present we show that the studied species present morphological and morphometric differences that can corroborate the hypotheses of Pita et al. [8] and Belintani et al. [7].

The evaluation of the eggs exochoria by SEM reveals an ornamentation with homogeneous geometric shapes, a common characteristic of the *Triatoma* genus as demonstrated by Barata [27]. In Triatomines, eggs have been explored in different approaches and have been shown to be an excellent taxonomic character [34,36,37,38]. The distinct sublayers of the chorion were first described by Beament [40] used eggs of *Rhodnius prolixus* Stål, 1859. Beament [40] makes observations about the composition of each layer and the process of formation of the geometric ornamentation present in the chorion. Barata [33] reiterate the histological descriptions by observing eggshells of ten *Rhodnius* Stål, 1859 species. Recently, the technique was used by Obara et al. [26] where it was possible to characterize the histological profile of eggs of six species of the *Triatoma*. In this study, it was possible to characterize the histological sections of the chorion of eggs of the four species for the first time. The photomicrographs of the histological sections did not allow the structures to be observed in great detail; however, it was possible to observe important characteristics, such as the four layers that form the egg chorion and their differences.

## 5. Conclusions

Bearing in mind that the four species studied are important for the current scenario of Chagas disease in South America, this study shows that the morphology and especially the morphometry of eggs are useful characters for differentiation between the studied species. Moreover, the female external genitalia by SEM are useful for taxonomy of these triatomines, especially visualized in the dorsal and ventral position. Finally, useful characters for the diagnosis and specific discrimination between *T.garciabesi*, *T. guasayana*, *T. patagonica* and *T. sordida sensu stricto* are presented, in addition, relevant systematic information is presented, confirming the current classification of these species.

## Figures and Tables

**Table 1:** Comparative morphology of the female external genitalia of *T. garciabesi, T. guasayana, T. patagonica*, and *T. sordida sensu stricto* in dorsal, posterior, and ventral view.

		Dorsal				Posterior	Ventral	
Species	The Shape of VII	The Shape of VIII	The Shape of IX	The Shape of X	The Shape of VIII	The Shape of X	Posterior Portions of the Connexiva of the VII	The Lines That Divide the Segments VIII and IX
*T. garciabesi*	straight lines, trapezoidal	trapezoidal	trapezoidal more evident	obtuse	VIII and IX segments are convex	semicircular	VII segment end in pointed tips	convex
*T. guasayana*	Straight lines, trapezoidal	trapezoidal	trapezoidal	obtuse	VIII and IX segments are flat	flat	VII segment end in rounded tips	Flat
*T. patagonica*	curved lines, trapezoidal	trapezoidal	trapezoidal	flat	VIII and IX segments are convex	Semicircular, curved elongated	VII segment end in pointed tips	Convex
*T. sordida sensu stricto*	curved lines, trapezoidal	trapezoidal	trapezoidal more evident	obtuse	VIII and IX segments are flat	Semicircular	VII segment end in rounded tips	flat

**Table 2:** Minimum (Min), maximum (Max), average ($\bar{x}$), and standard deviations (SD) of the length and opening of the perimeter of the operculum ofof length and opening of the perimeter of the operculum. *T. garciabesi*, *T. guasayana*, *T. patagonica*, and *T. sordida sensu stricto*.

Species	Length				Opening of the Perimeter of the Operculum			
	Min	Max	$\bar{x}$	SD	Min	Max	$\bar{x}$	SD
*T. garciabesi*	1.59	1.87	1.70	0.06	1.29	1.61	1.47	0.10
*T. guasayana*	1.33	1.79	1.60	0.09	1.19	1.57	1.42	0.08
*T. patagonica*	1.69	1.86	1.77	0.05	1.65	2.03	1.55	0.07
*T. sordida ss*	1.60	1.83	1.72	0.05	1.23	1.70	1.83	0.10

**Table 3:** Results of Turkey’s post-hoc test for length (bold) opening of the perimeter of the operculum (italic).

Species	*T. garciabesi*	*T. guasayana*	*T. patagonica*	*T. sordida s.s*
*T. garciabesi*	0.00	*** < 0.001	*** < 0.001	*** < 0.001
*T. guasayana*	*** < 0.001	**0.00**	*** < 0.001	*** < 0.001
*T. patagonica*	*** < 0.001	*** < 0.001	**0.00**	*** < 0.001
*T. sordida s.s*	***ns***	*** < 0.001	* < 0.033	**0.00**

*ns*: not significant, ***, * significant.
